# Comparison of diagnostic efficacy of ^18^F-FDG PET/CT and ^68^Ga-DOTANOC PET/CT in ectopic adrenocorticotropic hormone syndrome

**DOI:** 10.3389/fendo.2022.962800

**Published:** 2022-09-23

**Authors:** Bing Zhang, Qiao He, Yali Long, Yuying Zhang, Xiaoyan Wang, Zhifeng Chen, Jianbo Liu, Xiangsong Zhang

**Affiliations:** Department of Nuclear Medicine, The First Affiliated Hospital of Sun Yat-Sen University, Guangzhou, China

**Keywords:** Cushing syndrome, ectopic adrenocorticotropic hormone syndrome, ^18^F-fluorodeoxyglucose, ^68^Ga-DOTANOC, positron-emission tomography/computed tomography

## Abstract

**Purpose:**

Fluorine-18 (^18^F)-fluorodeoxyglucose (FDG) positron emission tomography/computed tomography (PET/CT) and gallium-68 (^68^Ga)-somatostatin analog (SSA) PET/CT imaging have been increasingly used in ectopic adrenocorticotropic hormone syndrome (EAS); however, the diagnostic efficacies of these two methods in patients with EAS remain unclear. Our study aimed to compare the diagnostic efficacies of ^18^F-FDG PET/CT and ^68^Ga-DOTANOC PET/CT in EAS.

**Methods:**

The clinical and imaging data of 68 patients with EAS who underwent ^18^F-FDG PET/CT and ^68^Ga-DOTANOC PET/CT examinations from December 2016 to April 2021 were analyzed retrospectively, and the diagnostic efficacies of these methods were compared.

**Results:**

In 37 cases, imaging was performed to locate the primary tumor lesion (localization group), and in 31 to evaluate tumor load or metastasis (staging group). Primary tumors were detected in 48.65% (18/37) of the localization group patients. According to scan-based analysis, the tumor lesion detection rates and false positive rates of ^18^F-FDG PET/CT imaging and ^68^Ga-DOTANOC PET/CT imaging were 18.92% vs. 45.95% (*p* < 0.05) and 21.62% vs. 2.70% (*p* < 0.05) respectively. For lesion-based analysis, the tumor lesion detection rates and false positive rates were 24.13% vs. 58.62% (*p >*0.05) and 31.04% vs. 3.45% (*p* < 0.05). In 90.32% (28/31) of the staging group patients, 286 of 292 lesions were confirmed as tumor lesions. Based on scan analysis, the detection rates and false positive rates of ^18^F-FDG PET/CT imaging and ^68^Ga-DOTANOC PET/CT imaging were 83.87% vs. 67.74% (*p* > 0.05) and 12.90% vs. 9.68% (*p* > 0.05) respectively. Based on lesion analysis, the detection rate and false positive rates were 93.84% vs. 54.80% (*p* < 0.05) and 1.37% vs. 1.03%(*p* > 0.05).

**Conclusion:**

^68^Ga-DOTANOC PET/CT imaging may be more suitable than ^18^F-FDG PET/CT for identifying the primary tumor in patients with EAS, while ^18^F-FDG PET/CT may be more advantageous than ^68^Ga-DOTANOC PET/CT for patients with suspected metastasis.

## Introduction

Cushing’s syndrome comprises adrenocorticotropic hormone (ACTH)-dependent Cushing’s syndrome, which accounts for 80% of cases, and ACTH-independent Cushing’s syndrome, which accounts for 20% of cases. Among ACTH-dependent Cushing’s syndrome, most cases are pituitary-dependent Cushing’s syndrome (Cushing’s disease, representing about 80%), and ectopic ACTH syndrome (EAS, representing about 20%) (1, 2). Ectopic secretion of corticotropin-releasing hormone (CRH) can also lead to ACTH-dependent Cushing’s syndrome, which is very rare.

Ectopic ACTH syndrome is mainly caused by excessive secretion of ACTH hormone by tumors outside the pituitary gland. This type of tumor is also called ectopic ACTH tumor and is widely distributed and highly heterogeneous. Medullary thyroid carcinoma is the main ectopic ACTH tumor in the neck, bronchial and thymic carcinoids are common in the chest, and small cell and large cell neuroendocrine carcinomas in the chest have also been reported (3–5). Neuroendocrine tumors of the stomach and pancreas as well as pheochromocytoma/paraganglioma are common in the abdominal cavity and retroperitoneum (3–5). Ectopic ACTH tumors can be divided into two types: dominant and recessive. The former is highly malignant, grows rapidly, and can easily be found, while the latter has low malignancy, grows relatively slowly, has a small tumor size, and is difficult to identify, and in some of them, the tumor source cannot be determined even after long-term follow-up (6). In view of the above characteristics, early detection and accurate staging of such tumors are extremely difficult.

At present, the traditional imaging methods used as first-line screening methods for locating ectopic ACTH tumors include ultrasonography, computed tomography (CT), and magnetic resonance imaging (MRI). The accuracy of these methods is not satisfactory in many cases, due to uncertainty about the tumor location. Functional imaging facilitates the detection of hidden tumors, can be used for further tumor burden assessment or staging after conventional imaging methods, and assists in inferring the biological characteristics of known tumors. Single-photon emission computed tomography/CT imaging with somatostatin analog indium-111 (^111^In)-labeled pentetreotide can show primary or metastatic neuroendocrine tumors (7, 8) expressing somatostatin receptor (SSTR) type 2 and type 5, and is widely used in this disease, but has a limited spatial resolution. Positron-emission tomography (PET)/CT has higher spatial resolution and sensitivity. Combined with multiple radiotracers, it can assist in tumor location, and the assessment of metabolic status and receptor expression level, with significant advantages.

For EAS, Fluorine-18 (^18^F)-fluorodeoxyglucose (FDG) PET/CT and gallium-68 (^68^Ga)-somatostatin analog (SSA) PET/CT imaging have shown certain advantages (9). However, the diagnostic efficacy of these two methods in patients with EAS and the appropriate choice of tracers for different patients remains unclear. To investigate these issues, the present study retrospectively analyzed the diagnostic efficacy of ^18^F-FDG PET/CT and ^68^Ga-DOTA-1-naphthylalanine 3-octreotide (DOTANOC) (a ^68^Ga-SSA imaging agent) PET/CT in patients with EAS.

## Materials and methods

### Research subjects

The clinical and imaging data of patients with EAS diagnosed in the First Affiliated Hospital of Sun Yat-Sen University Hospital from December 2016 to April 2021 were retrospectively analyzed. The patients’ diagnosis was made with reference to practice guidelines and routine diagnostic procedures of endocrinology societies (3, 10). The inclusion criteria were as follows: clinical diagnosis of EAS, necessitating locating the tumor or evaluating the tumor load (or staging); an interval between ^18^F-FDG PET/CT and ^68^Ga-DOTANOC PET/CT examinations of less than 2 weeks. Patients were excluded if the clinical diagnosis was Cushing’s disease; if no imaging examination was performed; if the interval between ^18^F-FDG PET/CT and ^68^Ga-DOTANOC PET/CT examinations was more than 2 weeks; if the follow-up data were incomplete or the follow-up period was less than 6 months. Among the 82 patients evaluated, 68 patients met the inclusion criteria.

### PET/CT imaging

A Gemini GXL 16 PET/CT scanner (Philips, Best, The Netherlands) and a Discovery MI PET/CT (GE, Boston, MA, USA) were adopted for PET/CT imaging, using the conventional 3-dimensional acquisition mode from the top of the skull to the middle femur. If head and neck lesions were suspected, it was necessary to add separate head and neck scanning. CT scanning parameters on the Gemini GXL 16 PET/CT were as follows: tube voltage 120 kV, tube current 100 mA, and slice thickness 5 mm. PET was collected in 6−7 positions (3 minutes per position). The ordered subset expectation maximization algorithm was used for PET image reconstruction. CT scanning parameters for the Discovery MI PET/CT were as follows: tube voltage 110 kV, tube current 80 mA, and slice thickness 3.75 mm. PET images were collected in 6−8 positions (2.0−2.5 minutes for each position). Block sequential regularized expectation maximization was adopted for the reconstruction of PET images. ^18^F-FDG and ^68^Ga-DOTANOC were prepared by our department. ^18^F-FDG was prepared by standard production technology and a commercial model system (Cyclone-10, Ion Beam Applications, Belgium), and ^68^Ga-DOTANOC was prepared by a ^68^Ge-^68^Ga generator (ITG GmbH, Oberding, Germany). Before ^18^F-FDG PET/CT examination, patients fasted for at least 6 hours, and it was confirmed that their fasting blood glucose level was lower than 7.8 mmol/L. For each patient, PET/CT examinations were performed 45−60 minutes after intravenous injection of ^18^F-FDG 5.55MBq/kg and ^68^Ga-DOTANOC 111-185MBq, and the interval between the two examinations was at least 24 hours.

### Image analysis

PET/CT images were jointly visually examined by two nuclear medicine doctors, jointly, who assessed abnormal radioactive uptake as well as the corresponding changes in morphology and structure and recorded the lesion location and number. At the same time, a region-of-interest (ROI) was delineated, and its maximum standardized uptake value (SUV_max_) was measured. Except for the physiological radioactive distribution area, other local abnormal radioactive concentrations or the radioactive uptake degree were higher than those of the surrounding normal tissues (based on the comparison between SUV_max_ in the ROI and that in the surrounding tissues), resulting in lesion-positive imaging diagnosis. Additionally, ratios of primary tumor SUVmax to mediastinal blood pool SUVmax (T/M) were calculated. The final diagnosis of the patients was confirmed according to laboratory examination, postoperative pathology or biopsy, imaging reexamination, and clinical follow-up for at least 6 months. The results of the Ki-67 proliferation index were also recorded for patients who obtained pathological examination results.

### Statistical analysis

Statistical analysis was performed using IBM SPSS 20.0 software (IBM Corp., Armonk, NY, USA). Quantitative data conforming to normal distribution were expressed as x̄ ± s, quantitative data not conforming to normal distribution were expressed as *M* (*P*_25_, *P*_75_), and qualitative data were expressed as frequency and percentage. According to the purpose of examination, patients were divided into a localization group and a staging group, and demographics and clinical characteristics between the two groups were calculated and compared using the Mann-Whitney U test or Chi-square test. Secondly, based on scanning and lesion analysis, the tumor lesion detection rate and false positive rate of ^18^F-FDG PET/CT and ^68^Ga-DOTANOC PET/CT was calculated and compared by McNemars’s test. *P* < 0.05 was considered to indicate a statistically significant difference.

## Results

### Patients

Sixty-eight patients with EAS, aged 19−72 [50.5 (41.5, 60.75)] years, with female patients accounting for 55.88%, underwent ^18^F-FDG PET/CT and ^68^Ga-DOTANOC PET/CT imaging. Among them, 37 cases were examined to detect or locate the tumor lesion (localization group), and 31 cases were examined to evaluate the tumor burden or metastasis (staging group). **Table 1** summarizes the patients’ demographics and clinical characteristics.

**Table 1:** Demographics, laboratory data, final diagnosis and tumor characteristics for ectopic ACTH syndrome patients.

	Localization group (n=37)	Staging group^a^ (n=31)	*P* value
Age(years)	48 (43, 59)	52 (41, 61)	0.980
Sex(F/M)	25/12	13/18	0.034
Serum cortisol (μg/dL)			
8 a.m.	31.80 (25.58, 40.70)	39.70 (23.90, 87.50)	0.371
4 p.m.	30.20 (25.38, 41.35)	43.27 (20.90, 74.60)	0.593
0 a.m.	26.05 (22.10, 34.43)	35.50 (21.40, 57.20)	0.264
Serum ACTH (pmol/L)	25.67 (20.88, 37.65)	31.10 (20.10, 69.30)	0.475
24h urine free cortisol (μg/24h)	2096.40 (712.95, 3630.93)	4017.00 (716.30, 7824.00)	0.162
Tumour source of overt EAS			
Anterior mediastinal/thymic carcinoid	7	14	—
Bronchial carcinoid	4	5	—
Pancreatic neuroendocrine tumor	4	6	—
Paraganglioma/pheoch-romocytoma	2	1	—
Medullary thyroid carcinoma	1	1	—
Olfactory neuroblastoma	0	2	—
Neuroendocrine carcinoma-unknown primary	0	2	—
Occult EAS	19	0	—
Maximum diameter of primary tumor (cm)	1.15 (0.98, 1.63)	4.10 (3.30, 5.40)	<0.0001
Ki-67 proliferation index	2%(1%, 8.5%)	15%(6.5%, 22.5%)	0.0001

a In the staging group, 11 of the 31 patients were patients after primary tumor resection.

### PET/CT results in the tumor localization group

Among the 37 patients in the localization group, ^18^F-FDG PET/CT and ^68^Ga-DOTANOC PET/CT combined imaging finally detected tumors in 18 patients (48.65%), all of which were isolated lesions, no metastatic lesions were found. These 18 patients were cured by surgery. Pathology showed anterior mediastinal/thymic carcinoids in 7 cases, bronchial carcinoids in 4 cases, pancreatic neuroendocrine tumors in 4 cases, paragangliomas/pheochromocytomas in 2 cases, and medullary thyroid carcinoma in 1 case (**Table 1**).

**Figure 1:**
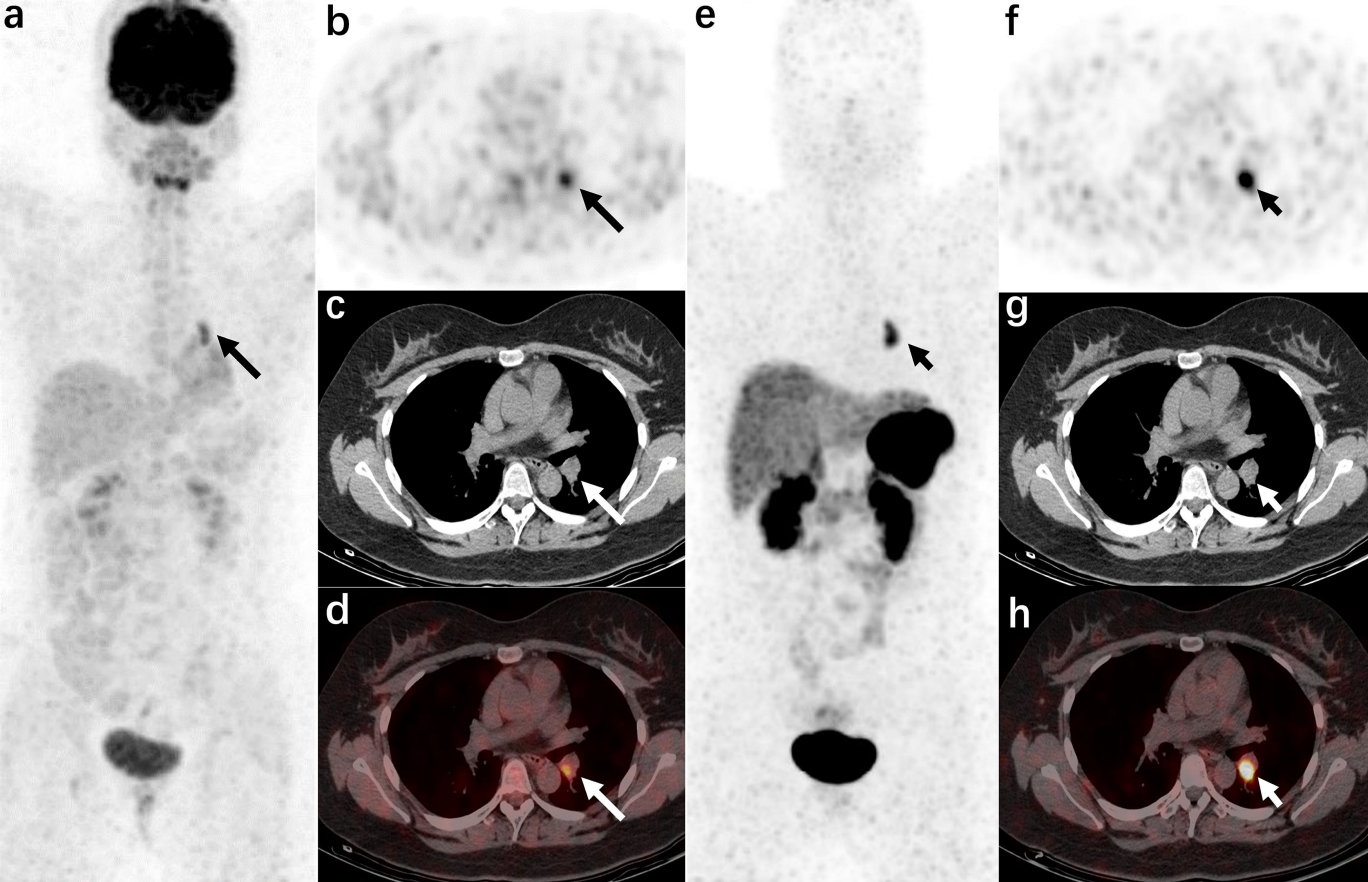
A 21-year-old woman was admitted to the hospital due to “a round face and weight gain for 7 months” (carcinoid of bronchi, case 21 in the localization group) The left hilar nodule was misdiagnosed as a lymph node by CT. ^18^F-FDG PET/CT [**(A)**: MIP image, **(B)**: axial PET, **(C)**: axial CT, **(D)**: axial PET/CT fusion image] and ^68^Ga-DOTANOC PET/CT [**(E)**: MIP image, **(F)**: axial PET, **(G)**: axial CT, **(H)**: axial PET/CT fusion image]. The radiotracer uptake increased in both examinations (the lesions in the ^18^F-FDG PET/CT images are shown by long arrows, and the lesions in the ^68^Ga-DOTANOC PET/CT images are shown by short arrows).

**Figure 2:**
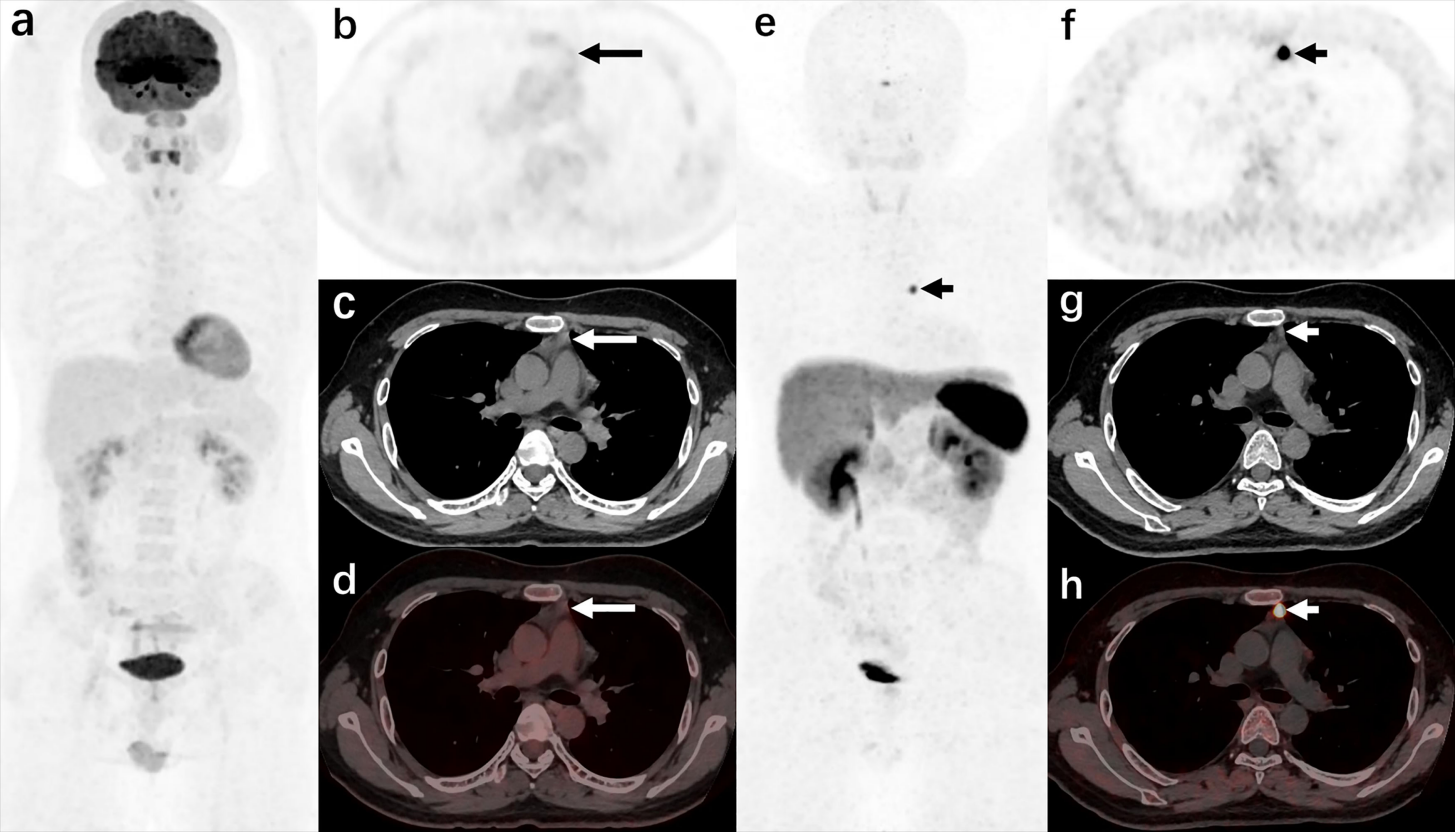
A 44-year-old man was admitted to the hospital due to “a round face and weight gain for 2 years, and elevated blood pressure for 1 year” (carcinoid of thymus, case 10 in the localization group) ^18^F-FDG PET/CT [**(A)**: MIP image, **(B)**: axial PET, **(C)**: axial CT, **(D)**: axial PET/CT fusion image] showed that there was no increase in radiotracer uptake in the anterior mediastinal nodule (long arrows); ^68^Ga-DOTANOC PET/CT **(E)**: MIP image, **(F)**: axial PET, **(G)**: axial CT, **(H)**: axial PET/CT fusion image] showed increased radiotracer uptake of the anterior mediastinal nodule.

In these overt EAS patients of the localization group, the detection rate of ^18^F-FDG PET/CT was 38.89% (7/18), while that of ^68^Ga-DOTANOC PET/CT was 94.44% (17/18). Moreover, 33.33% (6/18) were both lesion-positive in two examinations (**Figure 1**), 61.11% (11/18) were lesion-positive in ^68^Ga-DOTANOC PET/CT imaging but negative in ^18^F-FDG PET/CT imaging (**Figure 2**), and only 5.56%(1/18) was lesion-negative in ^68^Ga-DOTANOC PET/CT imaging but lesion-positive in ^18^F-FDG PET/CT imaging. According to scan-based analysis, the tumor lesion detection rate of ^68^Ga-DOTANOC PET/CT imaging was higher than that of ^18^F-FDG PET/CT imaging. In addition, the false positive rate of ^68^Ga-DOTANOC PET/CT imaging was lower than that of ^18^F-FDG PET/CT imaging based on the scan analysis and lesion analysis (**Table 2**). For the semi-quantitative analysis, the T/M ratio of the localization group tumors on ^68^Ga-DOTANOC PET/CT imaging was also higher than that of ^18^F-FDG PET/CT imaging(10.75(5.90,16.76) VS 1.62(1.82,3.64); p=0.003).

**Table 2:** PET/CT scan-based and lesion-based diagnostic results for patients with ectopic ACTH syndrome in the tumour localization group.

	Scan-based analysis (n=37)			Lesion-based analysis (n=29)		
	^18^F-FDG PET/CT	^68^Ga-DOTANOCPET/CT	*p*value	^18^F-FDGPET/CT	^68^Ga-DOTANOCPET/CT	*p* value
Detection rate	18.92% (7/37)	45.95% (17/37)	0.006	24.13% (7/29)	58.62% (17/29)	0.832
False positive rate	21.62% (8/37)	2.70% (1/37)	0.039	31.04% (9/29)	3.45% (1/29)	0.022

No tumor lesions were found in the remaining 19 occult EAS patients of the localization group, therefore, the actual detection rates of ^18^F-FDG PET/CT and ^68^Ga-DOTANOC PET/CT in all the localization group patients were not high (18.92% and 45.95%, respectively). All of these occult EAS patients received drug therapy (mifepristone or metyrapone). Three patients died of complications (2 cases of septic shock and 1 case of myocardial infarction). Only 6 patients finally underwent bilateral adrenalectomy, and the rest remain under follow-up, with no tumor found to date.

In the localization group, there were 8 patients with false-positive lesions in ^18^FDG PET/CT (**Supplementary Table 1**, cases 3, 7, 14, 16, 25, 33, 35 and 36), among whom 7 patients were confirmed by follow-up or puncture biopsy pathology to have pulmonary inflammation (cases 3, 7, 16, 25, 33, 35 and 36), and the remaining 1 patient (case 14) was confirmed by postoperative pathology to have reactive hyperplasia of the mediastinal lymph nodes. One patient (case 12) was false positive in ^68^Ga-DOTANOC PET/CT imaging and was confirmed to have follicular thyroid adenoma by postoperative pathology. In addition, 1 patient (case 2) was found to have anterior mediastinal nodules by CT, and both ^18^FDG PET/CT and ^68^Ga-DOTANOC PET/CT images were negative. The patient chose surgery and was pathologically confirmed to have thymoma (AB type).

### PET/CT results in the tumor staging group

There were 31 patients in the staging group, 11 of whom were patients who had undergone primary tumor resection(**Supplementary Table 2**). This group included 14 cases of anterior mediastinal/thymic carcinoid (5 cases after primary tumor resection), 5 cases of bronchial carcinoid (3cases after primary tumor resection), 6 cases of pancreatic neuroendocrine tumor(1 case after primary tumor resection), 1 case of paraganglioma/pheochromocytoma (after primary tumor resection), 1 case of medullary thyroid carcinoma (after primary tumor resection), 2 cases of olfactory neuroblastoma, and 2 cases of neuroendocrine carcinoma-unknown primary. ^18^F-FDG PET/CT and ^68^Ga-DOTANOC PET/CT finally detected tumor lesions in 90.32% (28/31) of the patients, among whom 26 patients (including 8 cases after primary tumor resection) showed metastatic lesions, 2 patients showed primary tumors without metastasis, and no recurrence and metastasis were found in the remaining patient who had undergone primary tumor resection (**Supplementary Table 2**).

**Figure 3:**
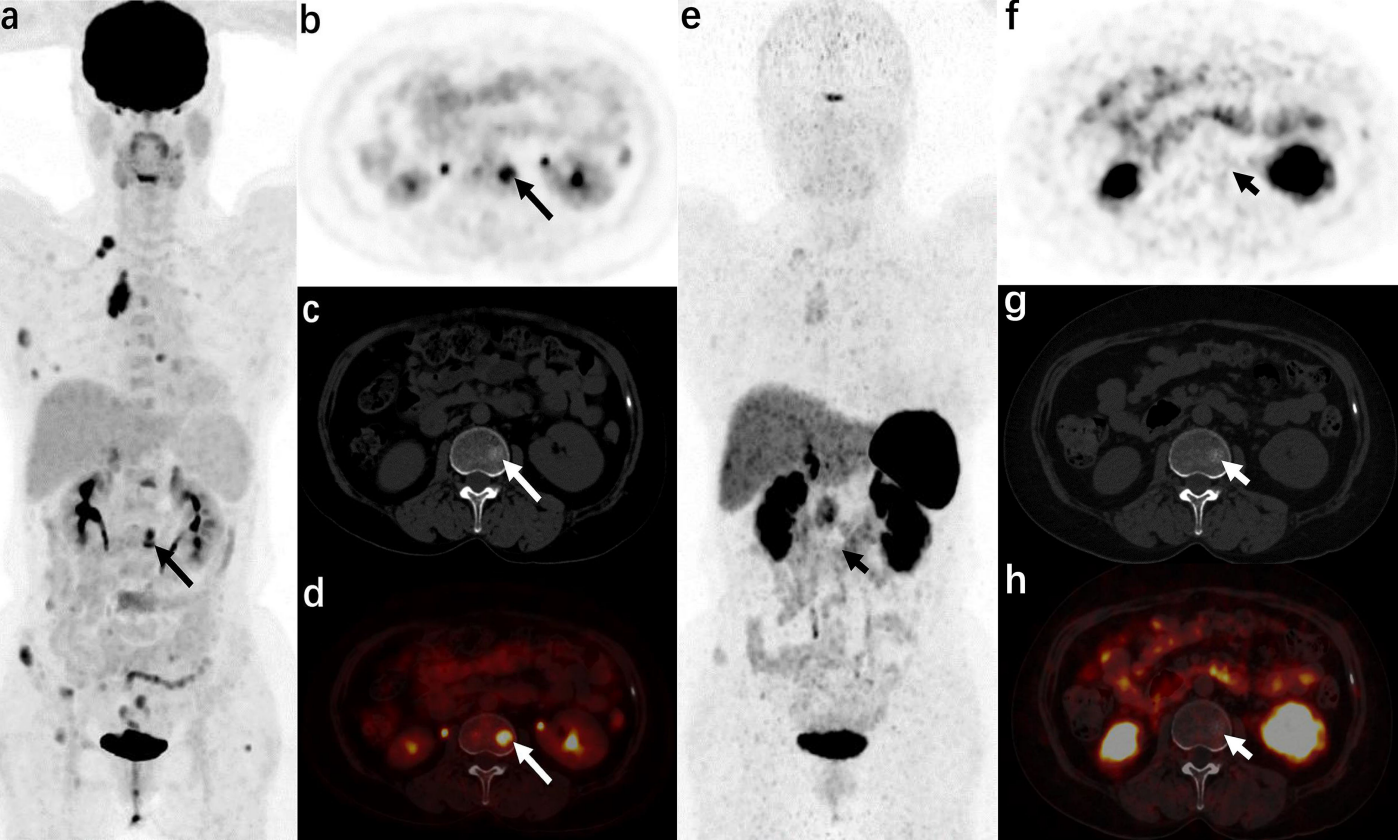
A 57-year-old woman was admitted to the hospital due to “blood pressure increased for 3 years and a full-moon face for 6 months” (atypical carcinoid of anterior mediastinum, case 14 in the staging group) ^18^F-FDG PET/CT [**(A)**: MIP image, **(B)**: axial PET, **(C)**: axial CT, **(D)**: axial PET/CT fusion image] showed that radioactive uptake increased in the anterior mediastinal tumor and multiple metastases (right supraclavicular lymph nodes, multiple ribs, multiple vertebrae, and adnexa, and right iliac bone), and axial images (b−d) show metastasis in the L2 vertebra (long arrows). ^68^Ga-DOTA NOC PET/CT [**(E)**: MIP image, **(F)**: axial PET, **(G)**: axial CT, **(H)**: axial PET/CT fusion image] only showed a slight increase in radiotracer uptake in the anterior mediastinal tumor and some metastases (right supraclavicular lymph node, some ribs, and right iliac bone), but no increase in radiotracer uptake was found in the metastases in some ribs, multiple vertebrae, and adnexa (short arrows).

In the staging group, 286 of 292 lesions were confirmed as tumor lesions by pathology or follow-up (see **Supplementary Tables 2**, **3** for details). According to lesion-based analysis, the detection rate of ^18^F-FDG PET/CT imaging was higher than that of ^68^Ga-DOTANOC PET/CT imaging (**Table 3**). Among the 286 confirmed tumor lesions, about 51.75% (148/286) lesions were positive in both examinations; 47.55% (126/286) were positive in ^18^F-FDG PET/CT but negative in ^68^Ga-DOTANOC PET/CT (**Figure 3**), while only 4.20% (12/286) were positive in ^68^Ga-DOTANOC PET/CT and negative in ^18^F-FDG PET/CT. For the semi-quantitative analysis, the T/M ratio of the staging group primary tumors did not reach statistical significance between ^18^F-FDG and ^68^Ga-DOTANOC PET/CT imaging. In addition, detection rate of ^18^F-FDG PET/CT imaging was also higher than that of ^68^Ga-DOTANOC PET/CT at different metastatic locations such as lymph nodes, liver and bones (**Table 4** and **Supplementary Table 3**).

**Table 3:** PET/CT scan-based and lesion-based diagnostic results for patients with ectopic ACTH syndrome in the tumor staging group.

	Scan-based analysis (n=31)			Lesion-based analysis (n=292)		
	^18^F-FDG PET/CT	^68^Ga-DOTANOCPET/CT	*p* value	^18^F-FDG PET/CT	^68^Ga-DOTANOCPET/CT	*p* value
Detection rate	83.87% (26/31)	67.74% (21/31)	0.180	93.84% (274/292)	54.80% (160/292)	<0.0001
False positive rate	12.90% (4/31)	9.68% (3/31)	1	1.37% (4/292)	1.03% (3/292)	1

**Table 4:** Detection rate of ^18^F-FDG PET/CT and ^68^Ga-DOTANOC PET/CT at different metastatic locations of the staging group.

	^18^F-FDG PET/CT	^68^Ga-DOTANOC PET/CT	*p* value
Lymph node	97.65% (83/85)	78.82% (67/85)	0.0004
Liver	98.97% (96/97)	38.14% (37/97)	<0.0001
Bone	94.12% (64/68)	38.24% (26/68)	<0.0001
Soft tissue (including muscles, pleura and pericardium)	72.22% (13/18)	100% (18/18)	–

Five patients had false-positive lesions (6 lesions in total) in the staging group. Among these, 1 patient (case 1 in the staging group) had a breast lesion. Both ^18^F-FDG PET/CT and ^68^Ga-DOTANOC PET/CT were “false-positive” (compared with ectopic ACTH tumors), but this breast lesion was finally confirmed as ductal adenocarcinoma by pathology. Two cases (case 4 and 31 in the staging group) with a false-positive result in ^18^FDG PET/CT imaging had pulmonary inflammation. Another patient (case 7 in the staging group) had a false-positive lesion in ^18^FDG PET/CT (confirmed by pathology as reactive hyperplasia of the lymph node) and a false-positive lesion in ^68^Ga-DOTANOCPET/CT (accessory spleen). The last case(case 30 in the staging group) also had an accessory spleen that showed a false-positive result in ^68^Ga-DOTANOC PET/CT imaging.

## Discussion

In this study, we compared the diagnostic efficacy of ^18^F-FDG PET/CT and ^68^Ga-DOTANOC PET/CT in EAS. We found that ^68^Ga-DOTANOC PET/CT imaging may be more suitable than ^18^F-FDG PET/CT for identifying the primary tumor in patients with EAS, while ^18^F-FDG PET/CT may be more advantageous than ^68^Ga-DOTANOC PET/CT for patients with suspected metastasis.

EAS is mainly caused by a variety of tumors that secrete ACTH. It has been reported in the literature that the tumors originate from various sites, including the neck, chest, abdomen, retroperitoneum, and some other rare sites (11). In this study, there were 49 patients with definite tumor lesions (including 11 postoperative patients and 2 cases of neuroendocrine carcinoma with unknown primary lesion), among which 61.22% were chest tumors (including 21 cases of anterior mediastinal/thymic carcinoid and 9 cases of bronchial carcinoid), essentially agreeing with previous reports (12–14), e.g., most of the ectopic tumors are distributed in the chest. Therefore, in the preliminary screening by imaging, it is necessary to pay particular attention to the examination of the chest. The proportion of abdominal tumors is second only to that of chest tumors (15–17) and was approximately 26.53% in this study (including 10 cases of pancreatic neuroendocrine tumors and 3 cases of pheochromocytoma/paraganglioma). Therefore, the abdomen, and particularly the pancreas, is also a key investigation site. Head and neck tumors accounted for a relatively low proportion (about 8.16%; including 2 cases of medullary thyroid carcinoma and 2 case of olfactory neuroblastoma) in the present study. In the absence of local symptoms, it is relatively difficult to diagnose such tumors, particularly when a plain CT scan is used as the primary screening method. In this study, Cushing’s syndrome caused by ACTH secreted by olfactory neuroblastoma was very rare; similarly, only a few cases have been reported in the literature (18, 19). Ectopic ACTH tumors have also rarely been reported in the mesentery, mammary glands, small intestine, heart, ovaries, and prostate (20–27). In this study, there were two cases of neuroendocrine carcinoma with extensive metastasis, but the primary lesion could not be identified. This might be because the primary tumor was relatively small or was concealed by a metastatic lesion. For instance, in case 10, a primary liver tumor with intrahepatic metastases is possible. Thus, although ectopic ACTH tumors have the same function (secreting ACTH, leading to Cushing’s syndrome), they have different organ locations and histological types, which greatly complicates the localization, diagnosis, and staging of lesions. Therefore, in addition to laboratory examination, comprehensive imaging examination is very important in their diagnosis and treatment.

CT and MRI are usually applied as first-line screening tools, while PET/CT is mainly applied for further tumor localization or evaluation. At present, ^18^F-labeled FDG is the most widely used tracer. This tracer mainly reflects the glycometabolism (anaerobic glycolysis) of tumor cells and has a positive correlation with tumor cell proliferation (28). ^18^F-FDG PET/CT has some value in the localization of ectopic ACTH tumors (29, 30). In addition, somatostatin receptor imaging has been widely reported in EAS (9, 31, 32). This mainly reflects the expression of the somatostatin receptor. ^111^In-labeled pentetreotide imaging has been widely used in the past, but due to its limited spatial resolution, ^68^GA-SSA PET/CT is gradually becoming the mainstream examination method. The widely used somatostatin receptor imaging tracers include ^68^Ga-DOTA-tyrosine 3-octreotide (DOTATOC), ^68^Ga-DOTA-1-naphthylalanine 3-octreotide (DOTANOC), and ^68^Ga-DOTA-D-phenylalanine 1-tyrosine 3-threonine 8-octreotide (DOTATATE). The difference in diagnostic efficacy of these three tracers in EAS is not clear at present. In this study, ^68^Ga-DOTANOC was used.

In this study, the overall tumor detection rate of PET/CT in patients in the localization group was less than 50%, which might be related to the high proportion of patients with occult ACTH tumors in our hospital. When we compared the two imaging agents in patients in the localization group with detected tumors(overt EAS patients), the detection rate using ^18^F-FDG was relatively low (38.89%, 7/18), while the detection rate using ^68^Ga-DOTANOC was relatively high (94.44%, 17/18). In addition, ^68^Ga-DOTANOC PET/CT had a higher tumor lesion detection rate based on scan analysis in all patients, and its T/M ratio (based on semi-quantitative analysis) was also higher than that of ^18^F-FDG PET/CT. This may mainly be due to the small lesions (the median maximum diameter was about 1.15 (0.98, 1.63) cm), low proliferation index [the median Ki-67 was 2%(1%, 8.5%)], and relatively high expression of somatostatin receptors in these tumors. Therefore, ^68^Ga-DOTANOC PET/CT may be more advantageous than ^18^F-FDG PET/CT in patients requiring detection of the primary tumor (particularly those in whom preliminary screening failed). Consistent with this study, a systematic review also demonstrated that the tumor detection rate of ^68^Ga-SSA PET/CT was higher than that of FDG(81.8% vs. 51.7%) (32). However, there were still 19 patients with occult lesions in the localization group in this study, in whom no tumor lesions were detected even by ^68^Ga-DOTANOC PET/CT. Varlamov et al. also concluded that the actual sensitivity of Ga-SSA PET/CT in patients with EAS (particularly in patients with occult lesions) is lower than that previously reported (31). Therefore, ^68^Ga-SSA PET/CT cannot completely solve the localization problem of ectopic ACTH tumors, but it has advantages over ^18^F-FDG. Additionally, a prospective study showed that although ^68^Ga-DOTATATE PET/CT had a higher detection rate than ^18^F-FDG PET/CT (75% vs 60%), the combination of two methods could achieve better diagnostic value than each alone (33). Thus, a recently published article (11) recommended that, for patients with EAS after the failure of preliminary screening by first-line imaging, ^68^Ga-DOTANOC PET/CT imaging should be performed first to screen for tumors, after which ^18^F-FDG PET/CT should be performed.

In contrast, in the staging group, the tumor proliferation index was relatively high [the median Ki-67 was 15%(6.5%, 22.5%)], and the number and detection rate of lesions by ^18^F-FDG PET/CT were markedly higher than those of ^68^Ga-DOTANOC PET/CT. Based on lesion analysis, the detection rate using FDG was higher than that using DOTANOC. Moreover, at different metastatic locations, for instance, lymph nodes, liver, and bone, the detection rate using FDG was also higher than that using DOTANOC. This may be because, in patients with metastatic ACTH tumors, the tumor showed strong invasion and had a high proliferation index, while the expression of the somatostatin receptor may be relatively low. At present, there are few comparative studies between ^18^F-FDG PET/CT and ^68^Ga-DOTANOC PET/CT in the same group of metastatic EAS tumors, and the conclusions obtained are inconsistent. For example, in a study by Kakade et al. (34), 5 patients (including 1 with multiple metastases of medullary thyroid carcinoma) were examined by ^18^F-FDG PET/CT and ^68^Ga-DOTATATE PET/CT at the same time, and the lesions were all positive in both examinations. In a study by Venkitaraman et al., 3 lung carcinoid patients (including 1 with mediastinal lymph node metastasis) were lesion-positive in ^68^Ga-DOTATOC PET/CT examination, but all were negative in ^18^F-FDG PET/CT examination (35). In addition, Liu et al. performed a head-to-head comparison of the diagnostic value of ^18^F-FDG and ^68^Ga-DOTATATE PET/CT and found that among the 8 patients with metastatic EAS tumors, 6 cases (primary tumor) were positive on ^18^F-FDG PET/CT imaging and 7 cases were positive on ^68^Ga-DOTATATE PET/CT imaging (33). Presumably, this was mainly related to the insufficient number of cases of this rare disease; thus, further studies with larger sample populations are required. According to this study, we believe that it might be a better choice to perform ^18^F-FDG PET/CT examination first for staging or metastatic patients.

There were some false-positive lesions in both ^18^F-FDG PET/CT and ^68^Ga-DOTANOC PET/CT imaging. In this study, there were 11 patients with a false-positive FDG result, which were related to inflammation. This is because FDG is a non-tumor-specific imaging agent; infectious diseases can also show increased glycometabolism, and patients with Cushing’s syndrome happen to be prone to infections. Therefore, the false positive rate of ^18^F-FDG PET/CT imaging was higher than that of ^68^Ga-DOTANOC PET/CT, and it should be considered when judging these imaging results. One patient was coincidentally found to have breast cancer lesions because of the positive results of ^18^F-FDG PET/CT and ^68^Ga-DOTANOC PET/CT imaging. Therefore, a second primary tumor still needs to be considered in image analysis. In addition, there were three other cases of false-positive lesions in ^68^Ga-DOTANOC PET/CT examination in this study: one was a thyroid adenoma lesion and the others were accessory spleen lesions. Yamaga et al. have also reported that ^68^Ga-DOTATATE has a relatively high uptake in non-Hodgkin lymphoma, meningioma, breast cancer, thyroid adenoma, and papillary carcinoma (36). Therefore, we should be alert to organs that are prone to false-positive uptake and pay attention to exclude a second primary tumor by also conducting other examinations.

Due to the possibility of slow proliferation of the tumor and the lack of expression of somatostatin, a patient with an anterior mediastinal nodule who was negative in both imaging examinations in this study and finally chose surgery was proven to have thymoma by pathology and did not show improvement of symptoms after the operation. Therefore, in the absence of sufficient evidence, it is not advisable to choose surgery blindly. In addition to the above two imaging agents, the literature reports that ^18^F-DOPA PET/CT imaging has a certain application value in EAS. For example, Acevedo-Báñez et al. used ^18^F-DOPA PET/CT to assist in locating an ectopic ACTH tumor in a patient with high FDG uptake in both lungs, which was confirmed by pathology (37). Schalin-Jäntti et al. reported a case of ectopic Cushing’s syndrome caused by large cell neuroendocrine carcinoma of the lung (38). ^68^Ga-DOTATOC PET/CT failed to locate the tumor, but ^18^F-DOPA PET/CT successfully located the primary tumor and its metastases (38). A prospective study by Zemskova et al. found that the tumor in 6 of 13 patients with EAS could be accurately located by ^18^F-DOPA PET/CT (7). Therefore, for patients negative in both ^18^F-FDG and ^68^Ga-DOTANOC PET/CT imaging, ^18^F-DOPA PET/CT imaging may play a further auxiliary role in diagnosis.

Our research had some limitations: first, as the number of cases was relatively small and a retrospective design was adopted, the results may not represent the overall situation of imaging in patients with EAS. Second, due to the selection bias in our hospital, there was a relatively large number of patients with occult ACTH tumors in the primary lesion group, which had a marked impact on the overall detection rate. Thirdly, in our retrospective study, the examination equipment used for the patients was not completely consistent. Considering the different performance and reconstruction methods of the two models of machines, this might have a certain impact on the examination results in theory. Finally, due to restriction by objective conditions, some patients did not undergo immunohistochemical detection of somatostatin receptor (data not shown), which made it impossible to confirm the expression of somatostatin receptor in ectopic ACTH tumors pathologically.

In conclusion, our study confirmed that ectopic ACTH tumors comprise many types of tumors with ACTH-secretion. They are mainly located in the chest and abdomen, and the most common tumors are thoracic carcinoid or atypical carcinoid types. For patients with ectopic ACTH tumors in whom first-line imaging screening failed, ^68^Ga-DOTANOC PET/CT cannot completely solve the problem of tumor localization, but it may be more suitable than ^18^F-FDG PET/CT in second-line approaches. Furthermore, ^18^F-FDG PET/CT has more advantages than ^68^Ga-DOTANOC PET/CT for patients with suspected metastasis. Our study provides preliminary guidance for PET/CT imaging examinations of ACTH tumor patients, but this needs to be confirmed by prospective studies with a larger number of cases.

## Data availability statement

The original contributions presented in the study are included in the article/**Supplementary Material**. Further inquiries can be directed to the corresponding authors.

## Ethics statement

The studies involving human participants were reviewed and approved by ethics committee of the First Affiliated Hospital of Sun Yat-sen University. The patients/participants provided their written informed consent to participate in this study. Written informed consent was obtained from the individual(s), and minor(s)’ legal guardian/next of kin, for the publication of any potentially identifiable images or data included in this article.

## Author contributions

XZ and JL, conception, design and supervision. BZ and QH, manuscript writing. BZ, QH, and YL, collection and analysis of data. YZ, XW, and ZC, analysis and interpretation of images. All authors contributed to the article and approved the submitted version.

## Funding

This work was supported by the Science and Technology Program of Guangzhou (202206010046).

## Conflict of interest

The authors declare that the research was conducted in the absence of any commercial or financial relationships that could be construed as a potential conflict of interest.

## Publisher’s note

All claims expressed in this article are solely those of the authors and do not necessarily represent those of their affiliated organizations, or those of the publisher, the editors and the reviewers. Any product that may be evaluated in this article, or claim that may be made by its manufacturer, is not guaranteed or endorsed by the publisher.
