# Supplementary material for: Comparison of diagnostic efficacy of 18F-FDG PET/CT and 68Ga-DOTANOC PET/CT in ectopic adrenocorticotropic hormone syndrome
Source: Front Endocrinol (Lausanne). 2022 Sep 23;13:962800. doi: 10.3389/fendo.2022.962800 (PMC9538925; doi:10.3389/fendo.2022.962800)
Supplement: Supplementary file 1 [file Table_1.doc]

Supplementary table 1 Demographics, imaging findings and final diagnosis for ectopic ACTH syndrome patients of localization group

| N | Age (Years)/Sex | Lesion location/Maximum lesion diameter | ^18^F-FDG PET/CT | ^68^Ga-DOTANOC PET/CT | Method of diagnosis | Final diagnosis | Ki-67 proliferation index |
| --- | --- | --- | --- | --- | --- | --- | --- |
| 1 | 32/F | No lesion found | Negative | Negative | Follow-up | No tumour found | ND |
| 2 | 30/F | Anterior mediastinal nodule /1.3cm | Negative | Negative | Operation | Benign thymoma (AB type)^a^ | — |
| 3 | 57/F | Left lower lung/1.9cm | False positive^b^ | Negative | Puncture biopsy | Inflammatory granuloma of the lung | ND |
| 4 | 48/F | Left lateral adrenal gland/1.0cm | Positive | Positive | Operation | Adrenal pheochromocytoma | 10% |
| 5 | 62/F | No lesion found | Negative | Negative | Follow-up | No tumour found |  |
| 6 | 58/F | Pancreatic head/1.8cm | Negative | Positive | Operation | Pancreatic NET（G1） | 2% |
| 7 | 60/F | Right thyroid lobe/1.6cm | Positive | Positive | Operation | Medullary thyroid carcinoma | 7% |
|  |  | Right middle lung lobe/2.6cm | False positive | Negative | Follow-up | Inflammation | ND |
| 8 | 45/F | No lesion found | Negative | Negative | Follow-up | No tumour found | ND |
| 9 | 46/M | Retroperitoneum /1.1 cm | Positive | Positive | Operation | Retroperitoneal paraganglioma | 8% |
| 10 | 44/M | Thymus/0.9cm | Negative | Positive | Operation | Typical carcinoid of the thymus | 2% |
| 11 | 39/F | No lesion found | Negative | Negative | Follow-up | No tumour found | ND |
| 12 | 58/F | Left thyroid lobe/1.2cm | Negative | False positive | Operation | Follicular thyroid adenoma | ND |
| 13 | 38/F | Thymus /0.7cm | Negative | Positive | Operation | Typical carcinoid of the thymus | 1% |
| 14 | 20/M | Anterior mediastinum /0.9cm | Positive | Positive | Operation | Atypical carcinoid of the anterior mediastinum | 10% |
|  |  | Mediastinal lymph nodes/1.0cm | False Positive | Negative | Follow-up | Reactive hyperplasia of lymph nodes | ND |
| 15 | 72/F | No lesion found | Negative | Negative | Follow-up | No tumour found | ND |
| 16 | 65/F | Right upper lung/3.2cm | False positive | Negative | Follow-up | Inflammation | ND |
| 17 | 47/F | No lesion found | Negative | Negative | Follow-up | No tumour found | ND |
| 18 | 50/F | Thymus /1.0cm | Positive | Negative | Operation | Atypical carcinoid of the thymus | 15% |
| 19 | 25/F | Anterior mediastinum/1.2cm | Negative | Positive | Operation | Typical carcinoid of the anterior mediastinum | 1% |
| 20 | 62/F | No lesion found | Negative | Negative | Follow-up | No tumour found | ND |
| 21 | 21/F | Left hilum /1.5cm | Positive | Positive | Operation | Typical carcinoid of bronchi | 1% |
| 22 | 43/M | Pancreatic tail /1.4cm | Negative | Positive | Operation | Pancreatic NET（G2） | 5% |
| 23 | 55/F | No lesion found | Negative | Negative | Follow-up | No tumour found | ND |
| 24 | 54/M | No lesion found | Negative | Negative | Follow-up | No tumour found | ND |
| 25 | 59/M | Left upper lung /1.1cm | Positive | Positive | Operation | Typical carcinoid of bronchi | 2% |
|  |  | Right upper lung/1.9cm | False positive | Negative | Follow-up | Inflammation | ND |
| 26 | 47/M | No lesion found | Negative | Negative | Follow-up | No tumour found | ND |
| 27 | 69/M | Right Middle lung lobe /1.7cm | Negative | Positive | Operation | Typical carcinoid of bronchi | 1% |
| 28 | 65/F | No lesion found | Negative | Negative | Follow-up | No tumour found | ND |
| 29 | 46/F | No lesion found | Negative | Negative | Follow-up | No tumour found | ND |
| 30 | 51/F | Thymus /0.8cm | Negative | Positive | Operation | Typical carcinoid of thymus | 2% |
| 31 | 46/M | Left lower lung/1.5cm | Negative | Positive | Operation | Typical carcinoid of bronchi | 1% |
| 32 | 40/M | Thymus /1.0cm | Negative | Positive | Operation | Atypical carcinoid of the thymus | 10% |
| 33 | 65/F | Right upper lung/1.1cm | False Positive | Negative | Follow-up | Inflammation | ND |
| 34 | 39/M | Pancreatic tail/1.7cm | Negative | Positive | Operation | Pancreatic NET（G2） | 5% |
| 35 | 61/M | Right upper lung/2.6cm | False Positive | Negative | Follow-up | Inflammation | ND |
| 36 | 53/F | Left lower lung/1.8cm | False Positive | Negative | Follow-up | Inflammation | ND |
| 37 | 67/F | Pancreatic head/2.0cm | Negative | Positive | Operation | Pancreatic NET（G1） | 1% |

ND, not done; M, male; F, female; FDG, fluorodeoxyglucose; PET/CT, positron emission tomography/computed tomography；NET, neuroendocrine tumour

^a^ The patient asked for surgical treatment, and the final pathological result was non-ectopic ACTH tumour.

^b^ All non-ACTH tumours with a positive imaging result were marked as False Positive in the imaging results column.
