# Supplementary material for: Comparison of diagnostic efficacy of 18F-FDG PET/CT and 68Ga-DOTANOC PET/CT in ectopic adrenocorticotropic hormone syndrome
Source: Front Endocrinol (Lausanne). 2022 Sep 23;13:962800. doi: 10.3389/fendo.2022.962800 (PMC9538925; doi:10.3389/fendo.2022.962800)
Supplement: Supplementary file 2 [file Table_2.doc]

Supplementary table 2 Demographics, imaging findings and final diagnosis for ectopic ACTH syndrome patients of staging group

| N | Age (Years)/  Sex | Primary tumour location/maximum diameter of primary tumour | ^18^F-FDG PET/CT ^a^ | ^68^Ga-DOTANOC PET/CT ^a^ | Metastatic lesions (including suspicious lesions) /number/maximum diameter | Method of  diagnosis | Final diagnosis | Ki-67 proliferation index |
| --- | --- | --- | --- | --- | --- | --- | --- | --- |
| 1 | 52/F | Postoperative atypical carcinoid of anterior mediastinum | ND | ND | Left supraclavicular lymph node/1/1.1 cm  Cardiac capsule/1/1.2 cm | Follow-up | Atypical carcinoid | 4% |
|  |  |  |  |  | Left mammary gland/1/1.0 cm | Puncture biopsy | Breast infiltrating ductal carcinoma | — |
| 2 | 65/M | Left lower lung/2.2 cm | Positive | Positive | Liver/5/2.9 cm | Liver puncture biopsy | Bronchi carcinoid | 6% |
| 3 | 49/M | Skull base/4.3 cm | Positive | Positive | Right cervical lymph nodes/1/2.9 cm | Surgery | Olfactory neuroblastoma | 15% |
| 4 | 27/M | Pancreatic head/5.5 cm | Positive | Positive | Retroperitoneal lymph nodes/1/1.1 cm  Liver/7/4.3 cm | Pancreas puncture biopsy | Pancreatic NET, G2 | 4% |
|  |  |  |  |  | Left upper lung/1/2.1 cm | Follow-up | Inflammation | ND |
| 5 | 62/F | Pancreatic head/3.2 cm | Positive | Negative | Peripancreatic lymph nodes/3/2.0 cm  Liver/10/8.5 cm | Liver puncture biopsy | Pancreatic NET, G3 | 50% |
| 6 | 46/M | Postoperative atypical carcinoid of thymus | ND | ND | Osteolytic bone metastasis/2/4.2 cm | Bone metastasis puncture biopsy | Atypical carcinoid | 8% |
| 7 | 23/M | Thymus/3.8 cm | Positive | Positive | No metastasis | Surgery | Atypical carcinoid | 10% |
|  |  |  |  |  | Mediastinal lymph nodes/1/1.4 cm  Accessory spleen/1.5 cm | Surgery and follow-up | Reactive hyperplasia of lymph nodes  Accessory spleen | ND |
| 8 | 43/M | Anterior mediastinum/2.3 cm | Positive | Negative | Osteogenic metastasis/11/3.6 cm | Bone puncture biopsy | Atypical carcinoid | ND |
| 9 | 56/F | Postoperative bronchial carcinoid in right lower lung | ND | ND | No metastasis | ND | ND | ND |
| 10 | 44/M | Primary lesion unknown | Unknown | Unknown | Hepatic portal and retroperitoneal lymph nodes/5/3.0 cm  Liver/15/12 cm  Osteogenic metastasis/1/2.0 cm | Liver puncture biopsy | Neuroendocrine carcinoma | 10% |
| 11 | 70/M | Right upper lung/2.8 cm | Positive | Negative | No metastasis | Surgery | Atypical carcinoid of bronchi | 7% |
| 12 | 33/M | Thymus/4.1 cm | Positive | Positive | Hepatic portal lymph nodes/3/2.9 cm  Liver/19/3.5 cm | Liver puncture biopsy | Atypical carcinoid | 15% |
| 13 | 54/F | Pancreatic head/3.3 cm | Positive | Negative | Liver/8/3.8 cm | Liver puncture biopsy | NET, G3 | 40% |
| 14 | 57/F | Anterior mediastinum/3.5 cm | Positive | Positive | Right supraclavicular lymph node/2/1.6 cm  Osteogenic metastasis 20/1.8 cm | Lymph node biopsy | Atypical carcinoid | 20% |
| 15 | 63/F | Pancreatic neck/3.0 cm | Positive | Positive | Left supraclavicular, right internal mammary, and retroperitoneal lymph nodes/9/2.6 cm  Liver/11/4.4 cm | Liver puncture biopsy | Pancreatic NET, G2 | 5% |
| 16 | 70/F | Pancreatic head/2.1 cm | Positive | Positive | Liver/8/3.6 cm | Liver puncture biopsy | Pancreatic NET, G2 | 5% |
| 17 | 59/M | Thymus/9.2 cm | Positive | Positive | Mediastinal lymph nodes/12/2.8 cm  Osteogenic metastasis/17/2.3 cm | Thymus puncture biopsy | Atypical carcinoid | 10% |
| 18 | 54/F | Postoperative pheochromocytoma of the right adrenal gland | ND | ND | Retroperitoneal lymph nodes/1/1.6 cm | Surgery | Pheochromocytoma | ND |
| 19 | 39/M | Postoperative atypical carcinoid of thymus | ND | ND | Right cervical, right supraclavicular, mediastinal, bilateral hilar, and right internal mammary lymph nodes/23/2.4 cm  Right pleura/1/1.5 cm  Osteogenic metastasis/10/2.1 cm | Lymph node puncture biopsy | Atypical carcinoid | 8% |
| 20 | 61/F | Anterior mediastinum/5.4 cm | Positive | Positive | Mediastinal and bilateral supraclavicular lymph nodes/12/3.9 cm | Puncture biopsy | Atypical carcinoid | 25% |
| 21 | 38/M | Postoperative atypical carcinoid of the thymus | ND | ND | Left chest wall/4/2.0 cm  Left pleura/3/2.1 cm | Chest wall puncture biopsy | Atypical carcinoid | 3% |
| 22 | 46/F | Thymus/3.6 cm | Positive | Negative | Left internal mammary lymph nodes/1/1.8 cm  Liver/14/4.3 cm | Liver puncture biopsy | Atypical carcinoid | 10% |
| 23 | 57/M | Postoperative medullary thyroid carcinoma | ND | ND | Right cervical lymph nodes/5/2.4 cm | Lymph node puncture biopsy | Medullary thyroid carcinoma | 30% |
| 24 | 19/M | Thymus/5.1 cm | Positive | Positive | Mediastinal and left supraclavicular lymph nodes/4/2.7 cm | Lymph node biopsy | Atypical carcinoid | 20% |
|  |  |  |  |  | Enostosis/1/1.5cm | Follow-up | Enostosis | ND |
| 25 | 50/M | Primary lesion unknown | Unknown | Unknown | Abdomino-pelvic masses/9/7.9cm  Osteogenic metastasis/2/1.6 cm | Puncture biopsy | Neuroendocrine carcinoma | 70% |
| 26 | 63/M | Skull base and ethmoid sinus /4.3 cm | Positive | Positive | No metastasis | Surgery | Olfactory neuroblastoma | 25% |
| 27 | 39/F | Postoperative bronchial atypical carcinoid in right lower lung | ND | ND | Osteolytic metastasis/2/2.3 cm | Puncture biopsy | Atypical carcinoid | ND |
| 28 | 47/F | Thymus/2.2 cm | Positive | Negative | Mediastinal lymph nodes/2/1.4 cm | Surgery | Atypical carcinoid | 15% |
| 29 | 41/F | Postoperative atypical carcinoid of the thymus | ND | ND | Osteogenic metastasis/3/3.1 cm | Puncture biopsy | Atypical carcinoid | ND |
| 30 | 56/M | Postoperative NET of the pancreatic tail | ND | ND | Accessory spleen/1.5 cm | Follow-up | Accessory spleen | ND |
| 31 | 62/M | Postoperative bronchial carcinoid in left lower lung | ND | ND | Left upper lung/1/1.9 cm | Follow-up | Inflammation | ND |

^a 18^F-FDG PET/CT and ^68^Ga-DOTANOC PET/CT columns indicate the imaging results of primary tumours. Postoperative patients are marked as ND (Not Done), and the patients whose primary lesions were unknown are marked as unknown.

M, male; F, female; ND, not done; FDG, fluorodeoxyglucose; PET/CT, positron emission tomography/computed tomography；NET，neuroendocrine tumour
