# Supplementary material for: Comparison of diagnostic efficacy of 18F-FDG PET/CT and 68Ga-DOTANOC PET/CT in ectopic adrenocorticotropic hormone syndrome
Source: Front Endocrinol (Lausanne). 2022 Sep 23;13:962800. doi: 10.3389/fendo.2022.962800 (PMC9538925; doi:10.3389/fendo.2022.962800)
Supplement: Supplementary file 3 [file Table_3.doc]

Table 3 Detection of metastatic lesions by ^18^F-FDG PET/CT and ^68^Ga-DOTANOC PET/CT in patients of the staging group

| N | Metastatic lesions detected by ^18^F-FDG PET/CT | | | | Metastatic lesions detected by ^68^Ga-DOTANOC PET/CT | | | |
| --- | --- | --- | --- | --- | --- | --- | --- | --- |
|  | Lymph node | Liver | Bone | Soft tissue  (including muscles, pleura and pericardium) | Lymph node | Liver | Bone | Soft tissue  (including muscles, pleura and pericardium) |
| 1 | 0 | 0 | 0 | 1 | 1 | 0 | 0 | 1 |
| 2 | 0 | 4 | 0 | 0 | 0 | 5 | 0 | 0 |
| 3 | 1 | 0 | 0 | 0 | 1 | 0 | 0 | 0 |
| 4 | 1 | 7 | 0 | 0 | 0 | 7 | 0 | 0 |
| 5 | 3 | 10 | 0 | 0 | 3 | 6 | 0 | 0 |
| 6 | 0 | 0 | 1 | 0 | 0 | 0 | 2 | 0 |
| 7 | 0 | 0 | 0 | 0 | 0 | 0 | 0 | 0 |
| 8 | 0 | 0 | 11 | 0 | 0 | 0 | 0 | 0 |
| 9 | 0 | 0 | 0 | 0 | 0 | 0 | 0 | 0 |
| 10 | 5 | 15 | 1 | 0 | 0 | 0 | 0 | 0 |
| 11 | 0 | 0 | 0 | 0 | 0 | 0 | 0 | 0 |
| 12 | 3 | 19 | 0 | 0 | 3 | 0 | 0 | 0 |
| 13 | 0 | 8 | 0 | 0 | 0 | 0 | 0 | 0 |
| 14 | 2 | 0 | 20 | 0 | 2 | 0 | 8 | 0 |
| 15 | 9 | 11 | 0 | 0 | 9 | 11 | 0 | 0 |
| 16 | 0 | 8 | 0 | 0 | 0 | 8 | 0 | 0 |
| 17 | 12 | 0 | 17 | 0 | 3 | 0 | 7 | 0 |
| 18 | 0 | 0 | 0 | 0 | 1 | 0 | 0 | 0 |
| 19 | 23 | 0 | 10 | 1 | 23 | 0 | 6 | 1 |
| 20 | 12 | 0 | 0 | 0 | 12 | 0 | 0 | 0 |
| 21 | 0 | 0 | 0 | 2 | 0 | 0 | 0 | 7 |
| 22 | 1 | 14 | 0 | 0 | 0 | 0 | 0 | 0 |
| 23 | 5 | 0 | 0 | 0 | 5 | 0 | 0 | 0 |
| 24 | 4 | 0 | 0 | 0 | 4 | 0 | 0 | 0 |
| 25 | 0 | 0 | 2 | 9 | 0 | 0 | 0 | 9 |
| 26 | 0 | 0 | 0 | 0 | 0 | 0 | 0 | 0 |
| 27 | 0 | 0 | 2 | 0 | 0 | 0 | 0 | 0 |
| 28 | 2 | 0 | 0 | 0 | 0 | 0 | 0 | 0 |
| 29 | 0 | 0 | 0 | 0 | 0 | 0 | 3 | 0 |
| 30 | 0 | 0 | 0 | 0 | 0 | 0 | 0 | 0 |
| 31 | 0 | 0 | 0 | 0 | 0 | 0 | 0 | 0 |
| Total | 83 | 96 | 64 | 13 | 67 | 37 | 26 | 18 |
